# JAK Inhibitors for Treatment of VEXAS Syndrome: A Systematic Review of 186 Cases

**DOI:** 10.1155/drp/9127126

**Published:** 2025-09-12

**Authors:** Saeed Bahramian, Patrick Fazeli, Arezou Rafati, Sardar Demokri, Huria Memari, Amirali Soheili, Farzad Esmaeili, Mohammad Pourmehdi Ardebili, Haniye Erfani, Seyed Mohammad Vahabi

**Affiliations:** ^1^School of Medicine, Isfahan University of Medical Sciences, Isfahan, Iran; ^2^Division of Biology & Medicine, Brown University, Providence, Rhode Island, USA; ^3^School of Medicine, Ilam University of Medical Sciences, Ilam, Iran; ^4^School of Medicine, Urmia University of Medical Sciences, Urmia, Iran; ^5^School of Medicine, Mazandaran University of Medical Sciences, Sari, Iran; ^6^Rajaie Cardiovascular Medical and Research Center, Iran University of Medical Sciences, Tehran, Iran; ^7^School of Medicine, Shahid Beheshti University of Medical Sciences, Tehran, Iran; ^8^School of Medicine, Golestan University of Medical Sciences, Gorgan, Iran; ^9^School of Medicine, Tehran Medical Sciences, Islamic Azad University, Tehran, Iran; ^10^Department of Dermatology, Razi Hospital, Tehran University of Medical Sciences, Tehran, Iran

**Keywords:** JAK inhibitors, Janus kinase, UBA1 mutation, VEXAS syndrome

## Abstract

**Objectives:** Vacuoles, E1 enzyme, X-linked, autoinflammatory, somatic (VEXAS) syndrome is an autoinflammatory disease with a wide spectrum of manifestations and no standard treatment. Janus kinase inhibitors (JAK-I) are small-molecule drugs that affect many molecular pathways. We aim to investigate the safety and efficacy of JAK-I in the treatment of VEXAS syndrome.

**Methods:** A systematic search was conducted using MeSH terms/keywords related to JAK-I and VEXAS syndrome through PubMed/Medline, Scopus, Web of Science, and Embase until July 6, 2025.

**Results:** We included 29 articles: 8 cohort, 8 case series, and 13 case reports. Our study includes data for 186 cases. The mean age was 69.64 years, and 83.33% were male. The most frequent manifestations were skin lesions (64.51%), fever (64.51%), arthritis and arthralgia (61.29%), lung involvement (31.72%), and venous thrombosis (24.19%). In general, 33.87% had a complete response, and 29.57% had a partial response. Ruxolitinib was used in 117 patients. Thirty-four out of 117 (29.06%) experienced complete to partial remission. Tofacitinib was used in 31 patients. About 29% of them showed complete to partial remission. Baricitinib was used in 25 patients; 12% had complete remission, and 16% had partial remission. Upadacitinib was used in 13 patients, which led to a complete remission in 38.46%. Filgotinib was used in four patients, leading to partial remission in one case. Among all, 36.55% showed adverse effects. Of these, eight were on Ruxolitinib, two on Tofacitinib, two on Baricitinib, and three on Upadacitinib.

**Conclusion:** JAK-I seems to be a promising treatment option with tolerable adverse effects for VEXAS syndrome.

## 1. Introduction

**Figure 1:**
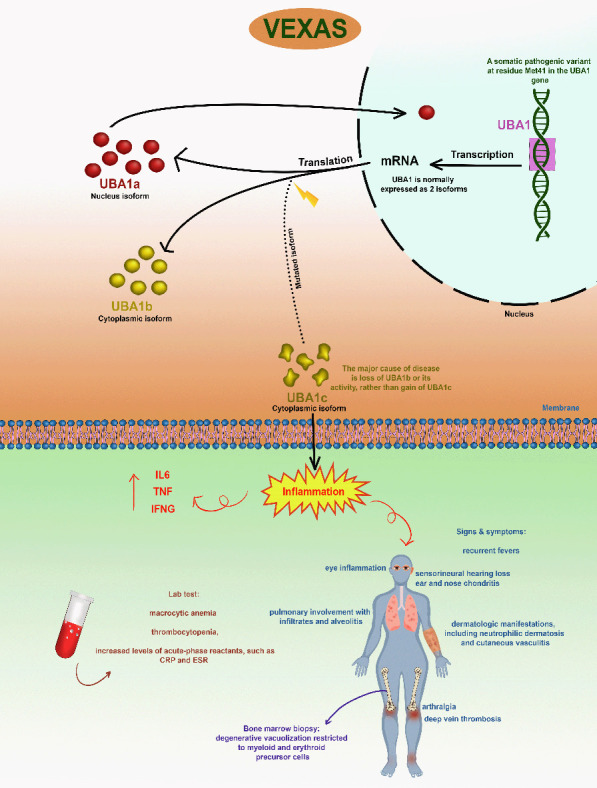
The impact of a somatic pathogenic mutation in the UBA1 gene and its clinical effects on humans (VEXAS) is illustrated graphically. A mutant cytoplasmic version of the UBA1 protein (UBA1c) is produced by a somatic pathogenic mutation at residue Met41 in the UBA1 gene. The UBA1 gene encodes the ubiquitin-activating enzyme E1, which has two primary isoforms: UBA1a and UBA1b. They serve a significant role in beginning ubiquitination, a fundamental mechanism for cellular control. DNA repair, gene expression, and cell cycle regulation are among the activities that are impacted by UBA1a's role in nuclear protein ubiquitination. Cytoplasmic protein ubiquitination, which affects immunological responses, signal transmission, and protein degradation, is the function of UBA1b. The disruption of normal cellular processes caused by this mutant isoform (UBA1c) results in increased inflammation (as indicated by raised levels of IL6, TNF, and IFNG) and a variety of clinical symptoms.

Vacuoles, E1 enzyme, X-linked, autoinflammatory, somatic (VEXAS) syndrome is a newly described disease. VEXAS is caused by a mutation in UBA1, which is an X-linked gene [1]. It usually occurs in older ages and is more prevalent in men [2]. Although this symptom is newly described, the common manifestations include recurrent fevers, pulmonary involvement, dermatologic manifestations, arthralgia, deep vein thrombosis, eye inflammation, and sensorineural hearing loss. Thrombocytopenia, elevated levels of acute-phase reactants, and macrocytic anemia can also be detected through laboratory testing [3] (Figure 1).

VEXAS pathophysiology is not fully elucidated yet, but the UBA1 mutation plays a major role in it. A mutant cytoplasmic version of the UBA1 protein (UBA1c) is produced by a somatic pathogenic mutation at residue Met41 in the UBA1 gene [4–7]. The disruption of normal cellular processes caused by this mutant isoform (UBA1c) results in increased inflammation (as indicated by raised levels of IL6, IL-1-beta, TNF, and IFNG), leading to a variety of clinical symptoms [8, 9]. Also, hematopoietic stem cells affect inflammatory pathways by myeloid differentiation and activation of these pathways [10].

Since VEXAS is a newly described disease and its reported cases are limited to cohorts of patients, there is no standard treatment for it. The most common treatments include glucocorticoids, disease-modifying antirheumatic drugs (DMARDs) such as hydroxychloroquine, and methotrexate; and also, hematopoietic stem cell transplant in some specific cases [11, 12].

Janus kinase inhibitors (JAK-I) are small-molecule drugs that affect many molecular pathways and have recently been approved or shown efficacy in many inflammatory and autoimmune diseases [13].

In this systematic review, we aim to investigate the safety and efficacy of JAK-I in the treatment of VEXAS syndrome.

## 2. Methods

### 2.1. Search Strategy

**Figure 2:**
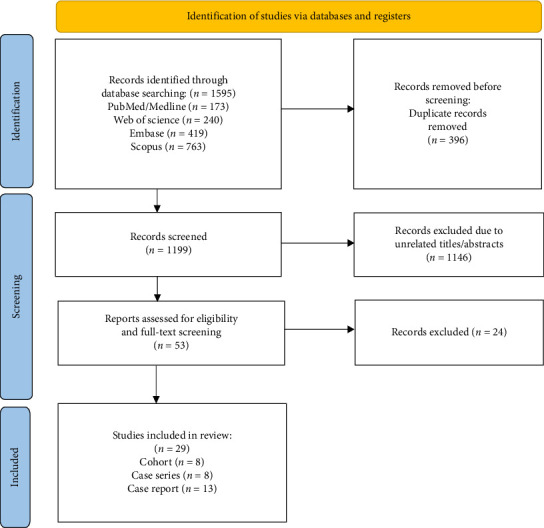
PRISMA flow chart of the number of studies identified and selected into the systematic review and meta-analysis.

A systematic search was conducted using MeSH terms/keywords related to JAK-I and VEXAS syndrome through PubMed/Medline, Scopus, Web of Science, and Embase until July 6, 2025. It follows the 2020 guidelines of the Preferred Reporting Items for Systematic Reviews and Meta-analyses (PRISMA) [14] (Supporting file 1) (see Figure 2).

### 2.2. Eligibility Criteria and Study Selection

The inclusion criteria were patients diagnosed with VEXAS syndrome who received at least one JAK-I. We excluded reviews, animal studies, and articles without enough data (Figure 2). Two authors used the National Heart, Lung, and Blood Institute (NHLBI) quality assessment tools for quality assessment of studies, except case reports (Supporting file 2).

### 2.3. Data Extraction

Six reviewers, divided into two groups, independently screened the articles and excluded unrelated ones. In case of disagreement, the corresponding author made the final decision. The extracted data included study characteristics, patient age, sex, symptoms, UBA1 mutation, other comorbidities, previous treatments, dosage, and duration of JAK-I, other concurrent medications, outcomes, and possible adverse effects. Also, seven articles had incomplete data and were not included in this study.

### 2.4. Outcome Definition

Outcome measurement varied in different studies. Some studies considered clinical symptom improvement to define the outcome, such as Al-Nusair et al. [15]; some considered both clinical symptoms and laboratory tests, such as Vitale et al. [16]; and some used imaging data, like Lechtenboehmer et al. [17], who used optical coherence tomography scans to compare the findings before and after treatment. Because of this variation, we considered complete remission as complete relief during therapy with JAK-I and resolution of clinical, laboratory, or imaging findings. Partial response was defined as the persistence of clinical, laboratory, and imaging findings with a remarkable decrease in their severity. Failure in treatment was defined as no changes in symptoms, laboratory tests, or imaging based on the author's description.

## 3. Results

Among all initially screened articles, we included 29 papers. Eight articles were cohort [11, 16, 18–23], 8 were case series [24–31], and 13 were case reports [15, 17, 32–42] (Tables 1, 2, 3).

Our study includes data for 186 cases. With a male predominance (155/186; 83.33%), the mean age was 69.64 years.

According to data collected from all included articles, the most frequent symptoms and signs were skin lesions (120/186; 64.51%), fever (120/186; 64.51%), joint involvement (114/186; 61.29%), lung involvement (59/186; 31.72%), and venous thrombosis (45/186; 24.19%). Almost all cases (181/186; 97.31%) had a UBA1 mutation, two patients were negative for mutation, and for three patients, data were not available.

The most common comorbidities in general were myelodysplastic syndrome (43/186; 23.11%) and monoclonal gammopathy (9/186; 4.83%). The most common medications that were used before JAK-I were glucocorticoids (43/186; 23.11%), methotrexate (29/186; 15.59%), mycophenolate mofetil (11/186; 5.91%), and azathioprine (8/186; 4.30%).

For disease control, most of the patients (126/186; 67.74%) received glucocorticoids besides JAK-I. Nearly all patients received only one JAK-I (184/186; 98.92%), except two patients who had to switch their medication due to incomplete response.

Among all cases, 63/186 (33.87%) had a complete response, 55/186 (29.57%) had a partial response, and 22/186 (11.82%) showed no response to treatment.

### 3.1. Ruxolitinib

Ruxolitinib was the most frequently used JAK-I to control VEXAS syndrome among all patients (117/186; 62.90%). In these patients, Ruxolitinib was used at a dose of 10–20 mg twice daily. In 31 patients (31/117; 26.49%), Ruxolitinib was used in combination with other medications. Concomitantly used medications included glucocorticoids (27/31; 87.09%) and DMARDs (4/31; 12.90%). Complete response was seen in 17 patients (17/117; 14.52%), 17 patients had a partial response (17/117; 14.52%), and 6 showed no response (6/117; 5.12%). Other patients' data were not specifically mentioned (77/117; 65.81%).

### 3.2. Tofacitinib

Tofacitinib was used in 31 patients (31/186; 16.66%). Ten patients received glucocorticoids, one received Mycophenolate mofetil, one received Azathioprine, and one patient received cyclosporine besides Tofacitinib. The Tofacitinib initial dose was 5 mg twice daily, and in some cases, the dose was increased up to 10 mg twice daily. Patients treated with Tofacitinib had complete remission (5/31; 16.12%), partial (4/31; 12.90%), and one had no remission (1/31; 3.22%). Data about outcomes were not available for 21 patients.

### 3.3. Baricitinib

Baricitinib was used in 25 patients (25/186; 13.44%). Seventeen patients received Baricitinib in combination with glucocorticoids (17/25; 68%) and DMARDs (2/25; 8%). Three patients had complete remission (3/25; 12%), four patients had a partial remission (4/25; 16%), and seven patients had no improvement (7/25; 28%). In 11 patients, there were no data about the outcomes of treatment.

### 3.4. Upadacitinib

Upadacitinib was used in 13 patients (13/186; 6.98%) with VEXAS syndrome, and in 8 patients, it was concomitantly used with glucocorticoids. The Upadacitinib mean dose was 15 mg daily. Among all cases, five had complete remission, two had partial remission, and data were not available for six cases.

### 3.5. Filgotinib

Filgotinib was used in four patients (4/186; 2.15%), with a dose of 200 mg/day. Partial response was observed in one patient (1/4; 25%). Data for the other three patients were not available.

### 3.6. Adverse Effects

Among all cases, 68 patients (68/186; 36.55%) showed adverse effects. Eight (8/68; 11.76%) were on Ruxolitinib, three on Upadacitinib (3/68; 4.41%), two on Tofacitinib (2/68; 2.94%), and two on Baricitinib (2/68; 2.94%). For other JAK-I, the side effects were not specifically mentioned (118/186; 63.44%). The side effects included infections (25/68; 36.76%), cytopenia (25/68; 36.76%), thrombosis (11/68; 16.17%), transient neutropenia (3/68; 4.41%), and minor systemic reaction after treatment (3/68; 4.41%).

## 4. Discussion

VEXAS syndrome is an inflammatory syndrome with a wide range of manifestations. In the absence of a standard treatment, studies showed various response rates in patients who received JAK-I.

JAK-I are a group of small-molecule drugs such as Ruxolitinib (JAK1-I and JAK2-I), Tofacitinib (JAK1-I), Baricitinib (JAK1-I and JAK2-I), Upadacitinib (JAK1-I), and Filgotinib (JAK1-I). JAK-Is can inhibit different inflammatory pathways through the inhibition of JAK 1, 2, 3, and TYK2. These subtypes of JAK and TYK affect a wide spectrum of cytokines and growth factors such as IL-2, IL-6, IL-12, IL-21, IFN, myeloproliferative leukemia (MPL), erythropoietin (EPO), granulocyte-macrophage colony-stimulating factor (GM-CSF), and thyroid peroxidase (TPO) [43]. This drug group showed promising efficacy in different dermatological conditions such as alopecia areata, morphea, and lichen planopilaris [44–46].

Although the etiology of VEXAS is not clear yet, we know some inflammatory cytokines, such as IL-6, IFN, and hematopoietic stem cells, play a role in the pathogenesis of the disease. Therefore, targeting the relevant inflammatory pathways with JAK-I could be more beneficial than using other drugs like IL-6 inhibitors, which only block one pathway [9, 43]. Also, JAK-I acts on a broad range of symptoms rather than merely suppressing them temporarily, as DMARDs do.

In the mentioned studies, JAK-I showed various response rates in cohorts to complete remission in case reports. This difference may be due to differences in the mechanisms of action of JAK-I. Ruxolitinib, which is a JAK1 and JAK2 inhibitor, showed more efficacy than the others. JAK2 affects different hematopoietic factors like GM-CSF, EPO, and MPL as well as a wide range of interleukins and interferons [18, 43].

In this review, 36.55% of patients showed adverse effects. Different studies showed that JAK-Is are generally not associated with increased cancer or cardiovascular risk; however, some studies suggest that using JAK-I in patients with underlying autoimmune or inflammatory disease should be done with caution because of their uncommon but serious side effects [47–50].

A significant concern in the management of VEXAS syndrome is the high incidence of venous thromboembolism (VTE), reported in 30%–50% of patients. This is particularly relevant when considering the use of JAK-I, as these agents have been associated with an increased risk of thrombotic events in certain populations [51, 52]. In our review, 11 cases of thrombosis were reported as adverse effects, the majority of which occurred in patients treated with Ruxolitinib. Although causality cannot be definitively established, the overlap of the inherent VEXAS-associated thrombotic risk with potential JAK-I-related prothrombotic effects is concerning. In the absence of formal guidelines, prescribers should remain vigilant and carefully evaluate the VTE risk when initiating JAK-I in VEXAS patients, particularly in those with a history of thrombotic events or additional prothrombotic risk factors. Consideration of thromboprophylaxis may be warranted in high-risk individuals, although this must be weighed against bleeding risk.

Another important dimension is the biological heterogeneity of VEXAS syndrome. Clinical manifestations and disease severity can vary widely among patients, with some exhibiting mild symptoms and low variant allele frequency (VAF), while others present with severe, steroid-dependent disease and high VAFs [29, 53]. This heterogeneity has therapeutic implications. JAK-Is, while effective in symptom control, do not appear to reduce VAF, suggesting they target inflammatory pathways without significantly affecting the underlying myeloid clone. On the other hand, agents like azacitidine may influence clonal hematopoiesis and potentially modify disease progression [54–56]. Understanding patient-specific factors, including VAF and clonal burden, is essential in guiding treatment decisions and evaluating the long-term benefit of symptom-directed therapy versus clonal-targeted strategies.

The results of this review are promising, although we should be aware of limitations. The major limitations of this review are the lack of large-scale trials and specified data in some of the included articles. Also, outcome measurement varied in different studies; however, we addressed this limitation by defining the outcomes in the methods section.

In conclusion, JAK-I seems to be a good and tolerable treatment option for the VEXAS syndrome, which has more efficacy than other current drugs. Also, the side effects are tolerable; however, larger studies with a long-term follow-up need to be done to shed more light on their long-term efficacy and safety.

## Figures and Tables

**Table 1:** Cohort studies on the use of Janus kinase inhibitors for the treatment of VEXAS syndrome.

#	Study Year Design	Number of patients Sex Age (median)	Signs and symptoms + UBA1 mutation	Comorbidities	Previous drugs	JAK-I + Concomitant drugs	Outcome Adverse effects (AEs)
1	Bourbon et al. [11] 2021 Cohort	1 Male 56	Fever, skin involvement, arthritis, pulmonary infiltrate, elevated CRP + Positive	MDS HBM	CSs, MTX, TCZ, ADA, azacytidine	Ruxo	PR AEs: None
		1 Male 68	Fever, skin involvement, arthritis, pulmonary infiltrate, elevated CRP + Positive	HBM	CSs, TCZ, ADA, azacytidine	Ruxo	PR AEs: None
		1 Male 53	Fever, skin involvement, arthritis, elevated CRP + Positive	HBM, FBM	CSs, TCZ	Tofa	PR AEs: None

2	Heiblig et al. [18] 2022 Retrospective cohort	30 Male [14] 67.9 (45.2–89.5)	Skin involvement [26], joints involvement [25], persistent fever [24], lung involvement [17], VTE [10] + Positive	MDS [12], atypical MDS/MPN [1], essential thrombosis [1]	CSs, TCZ, MTX, anakinra, 5-azacytidine	Ruxo [12], Tofa [11], Bari [4], Upa [3]	Ruxo: CR [6], PR [2], NR [2]; other JAKIs: CR [2], PR [1] AEs: Transient neutropenia [3] with Ruxo, VTE [2] with Ruxo and [4] with other JAKIs, herpetic keratitis [1] with Upa

3	Casal Moura et al. [19] 2023 Retrospective cohort	7 Male 68	Respiratory symptoms, skin lesions, fever, macrocytic anemia, chondritis, VTE, bone marrow vacuoles in early erythroid and granulocytic precursors + Positive	MM, MDS, PAN,RA, sweet syndrome, DRESS	Biologic agents (42%), conventional agents (58%)	Tofa [2], Ruxo [1], Upa [2], Bari [2] +GCs [7]	CR AEs: None

4	Gurnari et al. [20] 2023 Cohort	6 Male 59	Macrocytic anemia, skin rash, chondritis, fever, pulmonary infiltrates, elevated CRP + Positive	MDS, MPN	Azacitidine	Ruxo [4], Bari [2] +DMARDs	CR with Ruxo [2], PR with Ruxo [2] & Bari [2] AEs: None

5	Hadjadj et al. [21] 2024 Retrospective cohort	78 Male 74	Constitutional (66), skin (63), arthritis [42], chondritis [23], pulmonary [31], ocular [21], VTE [27] + Positive	MDS [18], MGUS [6]	MTX [13], MMF [4], AZA [3], CP [3]	Ruxo (68), Tofa [7], Bari [2], Upa [1] +GCs (72)	CR [26], PR [18], NR [4] AEs: Infection [18], cytopenia [18], thrombosis [5], minor systemic [3]

6	Vitale et al. [16] 2025 Cohort	15 NA 66.4	Fever, skin involvement, orbital involvement, arthritis, chondritis, vessel involvement, anemia + Positive	Relapsing polychondritis, sweet syndrome, polyarteritis nodosa, spondyloarthritis, SLE, polymyalgia rheumatic, Hodgkin's lymphoma, BLL, MDN, MPN, MGUS IgG kappa	DMARDs, GCs [15], colchicine	Ruxo [7], Tofa [3] Filg [3], Bari [2], Upa [2] + Prednisone [15]	CR [4], PR [8], NR [4] AEs: gut perforation (1 on Bari), Legionnaires' disease (1 on Upa), infectious pneumonia [1], sepsis-DIC (1 on Bari), neutropenia and thrombocytopenia (1 on Ruxo), insomnia (Tofa)

7	Al-Hakim et al. [22] 2025 Cohort	11 Male 67	Fever [9], skin involvement [10], vasculitis [5], arthritis [4], orbital involvement [4], chondritis [2], macrocytic anemia [9] thrombocytopenia [7] + Positive	MDS [3], MGUS [2]	MTX [4], AZA [2], MMF [2]	Bari + GCs [11]	PR [2], NR [6] AEs: Infection [2], cytopenia [1]

8	Wolff et al. [23] 2025 cohort	7 Male 67.5	Constitutional, skin involvement, chondritis, arthritis, vasculitis + Positive	MDS, LPD, MGUS	NA	Ruxo [4], Upa [1], Tofa [2] + GCs [7], CSA	CR: Ruxo [4], Tofa [1], Upa [1]; NR: Tofa [1] AEs: None

*Note:* CSs: corticosteroid, TCZ: Tocilizumab, MDS: Myelodysplastic syndrome, MTX: methotrexate, ADA: adalimumab, Ruxo: Ruxolitinib, Tofa: Tofacitinib, Bari: Bari, Upa: Upadacitinib, VTE: venous thromboembolism, MPN: myeloproliferative neoplasm, JAK-I: Janus kinase inhibitor, PAN: polyarteritis nodosa, GCs: glucocorticoids, DMARDs: disease-modifying antirheumatic drugs, CP: cyclophosphamide, LPD: lymphoproliferative disease, MGUS: monoclonal gammopathy, MMF: mycophenolate mofetil, AZA: azathioprine, Filg: filgotinib, and CSA: cyclosporine.

Abbreviations: BLL, B-lymphoblastic leukemia/lymphoma; CR, complete response; CRP, C-reactive protein; DIC, disseminated intravascular coagulation; DRESS, drug reaction with eosinophilia and systemic symptoms; FBM, fibrosis of bone marrow; HBM, hypercellular bone marrow; MM, multiple myeloma; NR, no response; PR, partial response; RA, rheumatoid arthritis; SLE, systemic lupus erythematous.

**Table 2:** Case series on the use of Janus kinase inhibitors for the treatment of VEXAS syndrome.

#	Study Year Number of patients	Sex Age	Signs and symptoms + UBA1 mutation	Comorbidities	Previous drugs	JAK-I + Concomitant drugs	Outcome Adverse effects (AEs)
1	Muratore et al. [24] 2022 One	Male 66	Fever, DVT, arthritis, dyspnea, skin involvement, chondritis + Positive	MDS with multilineage dysplasia	PRZ, MTX, Azathioprine	Upadacitinib 15 mg/day + PRZ	Complete remission AEs: none

2	Salehi et al. [25] 2023 Three	Male 72	Fever, DVT, urticaria, pancytopenia + Positive	Recurrent SIRS and IEOI, macrocytic anemia, MSGU	PRZ	Tofacitinib 5 mg BD + PRZ	Complete remission AEs: none
		Male 69	Fever, pruritus, anorexia, and weight loss, chondritis, DVT, PTE, skin involvement, elevated CRP, pancytopenia, pulmonary disease + Positive	Prostate adenocarcinoma	PRZ	Tofacitinib 5 mg BD + PRZ	Partial remission AEs: Delirium, respiratory distress, raised inflammatory markers, pancytopenia
		Male 72	Cutaneous reactions, lymphadenopathy, pancreatitis, dacryoadenitis, VTE, constitutional symptoms, progressive pancytopenia + Positive	Orbital inflammation, ILD, MDS, RBC-TDA	PRZ, MTX, AZA, MMF	Tofacitinib 5 mg BD + PRZ	Partial remission AEs: none

3	Diral et al. [26] 2024 Three	Male > 60	Cytopenia, orbital pseudotumor + NA	CCUS	GCs, CSA	Ruxolitinib + GCs	Partial remission AEs: none
		Male > 60	Cytopenia, lung inflammation, cutaneous vasculitis + NA	ICUS	GCs, TCZ	Ruxolitinib + GCs	Partial remission AEs: none
		Male > 60	Cytopenia, lung inflammation, ear and nose chondritis + NA	NA	GCs	Ruxolitinib + GCs, 5-azacitidine	Partial remission AEs: none

4	Kreutzinger et al. [27] 2024 Three	Male 60	Peripheral DVT, dyspnea, muscle weakness, Raynaud-like symptoms, fever + Positive	MDS, macrocytic hyperchromic anemia	MTX, LEF, PRZ	Ruxolitinib 20 mg BD + PRZ, azacytidine	No remission AEs: dizziness, headache, fever, constipation
		Male 70	Fever, dyspnea, pulmonary involvement, recurrent sterile parotitis, DVT + Positive	ILD, MDS	PRZ, azacitidine	Ruxolitinib 20 mg BD	Partial remission AEs: Mild decrease in Hb
		Male 80	Intermittent fever, weight loss, a history of skin rashes and polyarthritis + Positive	Polyarticular CPPD	PRZ, anakinra	Ruxolitinib 20 mg BD + PRZ	Complete remission AEs: none

5	Mishra et al. [28] 2024 Two	Male 68	Tender nonpruritic rash, recurrent fever, inflammatory arthritis + Negative	EN, CMML, macrocytic anemia	Prednisone, HCQ, MTX, MMF	Upadacitinib 15 mg daily + PRZ Switched to Ruxolitinib 10 mg BD	Partial remission with Upadacitinib; complete remission with Ruxolitinib AEs: none
		Male 77	Persistent pruritus, intermittent skin rash + Negative	CIU, BP, ACD, MDS, macrocytic anemia	OMA, PRX, RTX, naltrexone, IVM, topical (AH, CSs, AFg), NB-UVB	Upadacitinib 15–30 mg daily	Complete remission AEs: Cytopenia

6	Álamo et al. [29] 2025 Two	Male 54	Fever, night sweats, weight loss, relapsing auricular and nasal chondritis, digital ischemia, septal panniculitis, widespread folliculitis, vestibular dysfunction with sensorineural hearing loss, arthritis in both ankles + Positive	NA	CSs, anakinra,	Ruxolitinib 20 mg BD	No remission AEs: none
		Male 64	Superficial venous thrombosis, bilateral auricular and nasal chondritis, polyarthritis, bilateral proptosis + Positive	NA	CSs, MTX	Ruxolitinib 15 mg daily	No remission AEs: none

7	Costa et al. [30] 2025	Male 60	Asthenia, erythematous skin lesions, arthritis, periorbital edema, fever, weight loss + Positive	Sweet's syndrome	PRZ, Tocilizumab	Upadacitinib 15 mg daily + PRZ	Partial remission AEs: pancytopenia

8	Mizes et al. [31] 2025	Male 71	Skin involvement, chondritis, elevated CRP +Positive	Macrocytic anemia	HCQ, MMF, TCZ, colchicine, MTX, dapsone	Ruxolitinib 10 mg daily + PRZ	Complete remission AEs: none

*Note:* MTX: methotrexate, MDS: myelodysplastic syndrome, PRZ: prednisone, MGUS: monoclonal gammopathy, MMF: mycophenolate mofetil, PTE: pulmonary thromboendarterectomy, ICUS: idiopathic and clonal cytopenia of undetermined significance, AZA: azathioprine, CPPD: calcium pyrophosphate deposition disease, CMML: chronic myelomonocytic leukemia, GCs: glucocorticoids, CSA: cyclosporine, LEF: lefulonamide, HCQ: hydroxychloroquine, OMA: omalizumab, PRX: paroxetine, IVM: ivermectin, AH: antihistaminic, CSs: corticosteroid, AFg: antifungal, NB-UVB: narrowband ultraviolet B, and RTX: Rituximab.

Abbreviations: ACD, allergic contact dermatitis; BP, bullous pemphigoid; CCUS, clonal cytopenia of undetermined significance; CIU, chronic idiopathic urticaria; CRP, C-reactive protein; DVT, deep vein thrombosis; EN, erythema nodosum; I-EOI, ischemic end-organ injury; ILD, interstitial lung disease; RBC-TDA, red blood cell transfusion-dependent anemia; and SIRS, systemic inflammatory response syndrome.

**Table 3:** Case reports on the use of Janus kinase inhibitors for the treatment of VEXAS syndrome.

#	Study Year	Sex Age	Signs and symptoms + UBA1 mutation	Comorbidities	Previous drugs	JAK-I + Concomitant drugs	Outcome Adverse effects (AEs)
1	Kao et al. [32] 2022	Male 50	Fever, fatigue, anorexia, pulmonary disease, mild splenomegaly + Positive	PAN, cytopenia, EBV-HLH	Dapsone, colchicine, MMF, MTX	Ruxolitinib 15 mg BD + Anakinra, PRZ, RTX	PR AEs: None

2	Loschi et al. [33] 2022	Male 60	Skin lesion + Positive	Macrocytic regenerative anemia	HCQ, thalidomide	Baricitinib then Ruxolitinib + MTX, IFX, GCs, anakinra, UST, CSA	Baricitinib: NR Ruxolitinib: PR HSCT + Ruxolitinib: CR AEs: none

3	Ronsin et al. [34] 2022	Male 72	Skin lesions, low Hb and platelet, high serum creatinine, proteinuria, hematuria, leukocyturia + Positive	AKI, AIN, CAD, LCV	PRZ, anakinra	Ruxolitinib + PRZ	PR AEs: none

4	Austestad et al. [35] 2023	Male 60	Night sweats, weight, skin lesions, pain in lower extremities + Positive	ET, PTE	PRZ, MTX	Ruxolitinib 10 mg/day + PRZ, anagrelide, anakinra	PR AEs: none

5	Bindoli et al. [36] 2023	Male 65	Fever, pulmonary disease, asthenia, DVT, tenosynovitis, chondritis, macrocytic anemia, elevated inflammatory markers + Positive	Prostatectomy, LCV, DVT, MDS	M-PRZ	Filgotinib 200 mg/day + M-PRZ	PR AEs: none

6	Fahmy et al. [37] 2023	Male 66	Skin involvement + Positive	NA	Topical halobetasol, topical tacrolimus, oral doxycycline, oral minocycline, oral HCQ, oral PRZ	Tofacitinib 5 mg BD increased to 10 mg BD	CR AEs: none

7	Mohammed et al. [38] 2023	Male 73	Skin involvement, fever, night sweats, HBM + Positive	HTN	M-PRZ	Baricitinib 2 mg/day + PRZ	CR AEs: none

8	Beecher et al. [39] 2024	Male 68	Recurrent dacryoadenitis, angioedema-like lesions, jaw aches, rash, elevated laboratory markers, splenomegaly + Positive	IAP, pulmonary diseases, MDS	PRZ, MTX	Tofacitinib 5 mg BD + Azathioprine, MMF	PR AEs: None

9	Langlois et al. [40] 2024	Male 80	Fever, generalized weakness, drowsiness, weight loss, skin rash, ear chondritis, elevated CRP, macrocytic anemia, confusion, headaches, cerebellar ataxia + Positive	Ischemic stroke, CNS vasculitis	PRZ	Ruxolitinib 15 mg BD +M-PRZ, Tocilizumab	PR AEs: none

10	Wang et al. [41] 2024	Male 66	Skin involvement, macrocytic anemia, fever, general weakness, night sweats + Positive	MDS, RGD	MTX, HCQ	Ruxolitinib 10 mg BD + PRZ	PR AEs: none

11	Al-Nusair et al. [15] 2025	Male 64	Persistent anemia, weight loss, fatigue, erythematous circumferential papules, low-grade fevers, myalgia, cough, episcleritis, chondritis + Positive	NA	PRZ	Ruxolitinib 10 mg daily + PRZ, insulin???	CR AEs: none

12	Kelly et al. [42] 2025	Male 63	Lymphoid hyperplasia, splenomegaly, migratory arthralgia, multifocal PTE, DVT, pancytopenia, recurrent oral and genital ulceration + Positive	Multifocal-PG, MDS, lobular panniculitis, neutrophilic vasculitis, HTN, GERD	PRZ, pantoprazole, rivaroxaban, telmisartan, metoprolol, alendronate, vitamin D, doxycycline, sulfamethoxazole, trimethoprim, MMF, MTX, AZA, IFX, adalimumab, Tocilizumab	Ruxolitinib	NR AEs: none

13	Lechtenboehmer et al. [17] 2025	Male 83	Visual field defect, fever, night sweats, arthritis, pneumonitis, chondritis + Positive	NA	GCs	Ruxolitinib 15 mg BD + Dexamethasone, bevacizumab	PR AEs: none

*Note:* MMF: mycophenolate mofetil, PAN: polyarteritis nodosa, EBV-HLH: Epstein–Barr virus-related hemophagocytic lymphohistiocytosis, MTX: methotrexate, PTE: pulmonary thromboembolism, MDS: myelodysplastic syndrome, HBM: hypercellular marrow, LCV: leukocytic vasculitis, PRZ: prednisone, RTX: Rituximab, HCQ: hydroxychloroquine, IFX: Infliximab, GCs: glucocorticoids, UST: Ustekinumab, CSA: cyclosporine, M-PRZ: methylprednisolone, HTN: hypertension, AZA: azathioprine, GCs: glucocorticoids, and GERD: gastroesophageal reflux disease.

Abbreviations: AIN, acute interstitial nephritis; AKI, acute kidney injury; CAD, coronary artery disease; CNS, central nervous system; CR, complete response; CRP, C-reactive protein; ET, essential thrombosis; HSCT, hematopoietic stem cell transplantation; IAP, idiopathic autoimmune pancreatitis; NR, no response; PR, partial response; PG, Pyoderma gangrenosum; and RGD, reactive granulomatous dermatitis.

## Data Availability

Data sharing is not applicable to this article, as no new data were created or analyzed in this study.

## Supporting Information

Supporting Information 1 Supporting file 1 shows the keywords and query we used for this systematic search.

Supporting Information 2 Supporting file 2 shows the quality assessment of included articles by the National Heart, Lung, and Blood Institute (NHLBI) quality assessment tools.

## References

[1] Beck D. B., Ferrada M. A., Sikora K. A. (2020). Somatic Mutations in UBA1 and Severe Adult-Onset Autoinflammatory Disease. *New England Journal of Medicine*.

[2] Hadjadj J., Nguyen Y., Mouloudj D. (2024). *OP0243 Efficacy and Safety of Targeted Therapies in VEXAS Syndrome: Retrospective Study From the FRENVEX*.

[3] Kobak S. (2023). VEXAS Syndrome: Current Clinical, Diagnostic and Treatment Approaches. *Intractable & Rare Diseases Research*.

[4] Poulter J. A., Collins J. C., Cargo C. (2021). Novel Somatic Mutations in UBA1 as a Cause of VEXAS Syndrome. *Blood*.

[5] Groen E. J., Gillingwater T. H. (2015). UBA1: At the Crossroads of Ubiquitin Homeostasis and Neurodegeneration. *Trends in Molecular Medicine*.

[6] Lambert-Smith I. A., Saunders D. N., Yerbury J. J. (2020). The Pivotal Role of Ubiquitin-Activating Enzyme E1 (UBA1) in Neuronal Health and Neurodegeneration. *The International Journal of Biochemistry & Cell Biology*.

[7] Majer D., Kujawińska M., Limanówka P., Sędek Ł. (2024). How Protein Ubiquitination Can Influence Cytokine Expression—Updated Review on Autoinflammatory VEXAS Syndrome. *Immunology*.

[8] Hernández-Rodríguez J., Mensa-Vilaró A., Aróstegui J. I. (2022). Paradigm Shift in Monogenic Autoinflammatory Diseases and Systemic Vasculitis: The VEXAS Syndrome. *Medicina Clínica*.

[9] Kosmider O., Possémé C., Templé M. (2022). VEXAS Syndrome is Characterized by Blood and Tissues Inflammasome Pathway Activation and Monocyte Dysregulation. *medRxiv*.

[10] Wu Z., Gao S., Gao Q. (2023). Early Activation of Inflammatory Pathways in UBA1-Mutated Hematopoietic Stem and Progenitor Cells in VEXAS. *Cell Reports Medicine*.

[11] Bourbon E., Heiblig M., Gerfaud Valentin M. (2021). Therapeutic Options in VEXAS Syndrome: Insights From a Retrospective Series. *Blood*.

[12] Diarra A., Duployez N., Fournier E. (2022). Successful Allogeneic Hematopoietic Stem Cell Transplantation in Patients With VEXAS Syndrome: A 2-Center Experience. *Blood Advances*.

[13] McLornan D. P., Pope J. E., Gotlib J., Harrison C. N. (2021). Current and Future Status of JAK Inhibitors. *Lancet*.

[14] Page M. J., McKenzie J. E., Bossuyt P. M. (2021). The PRISMA 2020 Statement: An Updated Guideline for Reporting Systematic Reviews. *Bmj*.

[15] Al-Nusair J., Lim O., Alhusari L. (2025). The Challenging and Unique Diagnosis of VEXAS Syndrome: A Case Report. *Journal of Investigative Medicine High Impact Case Reports*.

[16] Vitale A., Caggiano V., Leone F. (2025). Efficacy and Safety Profile of Biotechnological Agents and Janus Kinase Inhibitors in VEXAS Syndrome: Data From the International AIDA Network VEXAS Registry. *Frontiers in Pharmacology*.

[17] Lechtenboehmer R., Mauschitz M. M., Holz F. G., Finger R. P., Wintergerst M. W. (2025). A Case of VEXAS Syndrome With Therapy Refractive Macular Involvement. *Canadian Journal of Ophthalmology*.

[18] Heiblig M., Ferrada M. A., Koster M. T. (2022). Ruxolitinib Is More Effective Than Other JAK Inhibitors to Treat VEXAS Syndrome: A Retrospective Multicenter Study. *Blood*.

[19] Casal Moura M., Baqir M., Tandon Y. K. (2023). Pulmonary Manifestations in VEXAS Syndrome. *Respiratory Medicine*.

[20] Gurnari C., Koster L., Baaij L. (2024). Allogeneic Hematopoietic Cell Transplantation for VEXAS Syndrome: Results of a Multicenter Study of the EBMT. *Blood Advances*.

[21] Hadjadj J., Nguyen Y., Mouloudj D. (2024). Efficacy and Safety of Targeted Therapies in VEXAS Syndrome: Retrospective Study From the FRENVEX. *Annals of the Rheumatic Diseases*.

[22] Al-Hakim A., Trikha R., Phyu Htut E. E. (2025). Treatment Outcomes in Patients With VEXAS Syndrome: A Retrospective Cohort Study. *The Lancet Rheumatology*.

[23] Wolff L., Caratsch L., Lötscher F. (2024). VEXAS Syndrome: A Swiss National Retrospective Cohort Study. *Swiss Medical Weekly*.

[24] Muratore F., Marvisi C., Castrignanò P. (2022). VEXAS Syndrome: A Case Series From a Single‐Center Cohort of Italian Patients With Vasculitis. *Arthritis & Rheumatology*.

[25] Salehi T., Callisto A., Beecher M. B., Hissaria P. (2023). Tofacitinib as a Biologic Response Modifier in VEXAS Syndrome: A Case Series. *International Journal of Rheumatic Diseases*.

[26] Diral E., Campochiaro C., Tomelleri A. (2024). Case Report: Cytopenias in VEXAS Syndrome-a WHO 2022 Based Approach in a Single-Center Cohort. *Frontiers in Immunology*.

[27] Kreutzinger V., Pankow A., Boyadzhieva Z. (2024). VEXAS and Myelodysplastic Syndrome: An Interdisciplinary Challenge. *Journal of Clinical Medicine*.

[28] Mishra R., Calabrese C., Jain A. G., Singh A. (2024). Association Between Myeloid Disorders and Adult Onset-Inflammatory Syndromes, Successful Treatment with JAK-inhibitors: Case Series and Literature Review. *Leukemia Research*.

[29] Álamo J. R., Torres L. M. d., Castaño-Díez S. (2025). Hypomethylating Agents for Patients With VEXAS Without Myelodysplastic Syndrome: Clinical Outcome and Longitudinal Follow‐Up of Vacuolization and UBA1 Clonal Dynamics. *British Journal of Haematology*.

[30] Costa A., Pilo F., Pettinau M. (2025). VEXAS Syndrome: Is It More a Matter of Inflammation or Hematopoietic Clonality? A Case Series Approach to Diagnosis, Therapeutic Strategies and Transplant Management. *Annals of Hematology*.

[31] Mizes A., Ash M. M., Richardson C. T. (2025). VEXAS Syndrome With p. Met41Leu UBA1 Gene Mutation Misdiagnosed as Tumid Lupus: A Series of 3 Cases. *JAAD Case Reports*.

[32] Kao R. L., Jacobsen A. A., Billington C. J. (2022). A Case of VEXAS Syndrome Associated With EBV-Associated Hemophagocytic Lymphohistiocytosis. *Blood Cells, Molecules, and Diseases*.

[33] Loschi M., Roux C., Sudaka I. (2022). Allogeneic Stem Cell Transplantation as a Curative Therapeutic Approach for VEXAS Syndrome: A Case Report. *Bone Marrow Transplantation*.

[34] Ronsin C., Benard L., Mourtada A., Perrin F., Boukerroucha Z. (2022). Acute Tubulointerstitial Nephritis Revealing VEXAS Syndrome. *Kidney International*.

[35] Austestad J., Madland T. M., Sandnes M., Haslerud T. M., Benneche A., Reikvam H. (2023). VEXAS Syndrome in a Patient With Myeloproliferative Neoplasia. *Case Reports in Hematology*.

[36] Bindoli S., Baggio C., Doria A., Bertoldo E., Sfriso P. (2023). JAK Inhibitors for the Treatment of VEXAS Syndrome. *Experimental Biology and Medicine*.

[37] Fahmy L. M., Schreidah C. M., Lapolla B. A., Magro C. M., Geskin L. J. (2023). VEXAS Syndrome Presenting as Refractory Cutaneous Kikuchi Disease-Like Inflammatory Pattern Responding to Tofacitinib. *JAAD Case Reports*.

[38] Mohammed T. O., Alavi A., Aghazadeh N. (2023). Vacuoles, E1 Enzyme, X‐Linked, Autoinflammatory, Somatic (VEXAS) Syndrome: A Presentation of Two Cases with Dermatologic Findings. *International Journal of Dermatology*.

[39] Beecher M. B., Tong J. Y., Halliday L. A., Hissaria P., Selva D. (2024). Recurrent Orbital Inflammation Associated With VEXAS Syndrome. *Orbit*.

[40] Langlois V., Curie A., Demas A. (2024). Central Nervous System Vasculitis in VEXAS Syndrome: A Rare Involvemen. *Clinical Neurology and Neurosurgery*.

[41] Wang C. X., Yokoyama C. C., Rosman I. S., Musiek A. C. (2024). Extensive Reactive Cutaneous Histiocytic Infiltrate Resembling Non-Langerhans Cell Histiocytosis as the Presenting Sign of Underlying Vacuoles, E1 Enzyme, X-linked, Autoinflammatory, Somatic Syndrome. *JAAD Case Reports*.

[42] Kelly G., Lobo Y., Godbolt A. (2025). Therapeutic Challenges in the Management of VEXAS Syndrome: A Case Report. *Australasian Journal of Dermatology*.

[43] Miot H. A., Criado P. R., de Castro C. C. S., Ianhez M., Talhari C., Ramos P. M. (2023). JAK-STAT Pathway Inhibitors in Dermatology. *Anais Brasileiros de Dermatologia*.

[44] Nasimi M., Abedini R., Ghandi N., Teymourpour A., Babaie H. (2024). Safety and Efficacy of Tofacitinib in 97 Alopecia Areata Patients. *Journal of Cosmetic Dermatology*.

[45] Ansari M. S., Vahabi S. M., Memari H., Hosseini F., Bahramian S., Etesami I. (2025). Use of the Oral Janus Kinase Inhibitor Tofacitinib in the Treatment of Morphea: A Retrospective Study. *Journal of the American Academy of Dermatology*.

[46] Nasimi M., Ansari M. S. (2024). JAK Inhibitors in the Treatment of Lichen Planopilaris. *Skin Appendage Disorders*.

[47] Russell M. D., Stovin C., Alveyn E. (2023). JAK Inhibitors and the Risk of Malignancy: A Meta-Analysis Across Disease Indications. *Annals of the Rheumatic Diseases*.

[48] Ingrassia J. P., Maqsood M. H., Gelfand J. M. (2024). Cardiovascular and Venous Thromboembolic Risk With JAK Inhibitors in Immune-Mediated Inflammatory Skin Diseases: A Systematic Review and Meta-Analysis. *JAMA dermatology*.

[49] Vahabi S. M., Bahramian S., Esmaeili F. (2024). JAK Inhibitors in Cutaneous T-Cell Lymphoma: Friend or Foe? A Systematic Review of the Published Literature. *Cancers*.

[50] Etesami I., Ansari M. S., Pourgholi E. (2025). Drug‐and Vaccine‐Induced Cutaneous T‐Cell Lymphoma: A Systematic Review of the Literature. *Journal of skin cancer*.

[51] Molander V., Bower H., Frisell T. (2023). Venous Thromboembolism With JAK Inhibitors and Other Immune-Modulatory Drugs: A Swedish Comparative Safety Study Among Patients With Rheumatoid Arthritis. *Annals of the Rheumatic Diseases*.

[52] Lowell J. A., Sharma G., Sultan K. (2024). Earlier Onset of Acute Venous Thromboembolism With Upadacitinib Compared With Tofacitinib During Janus Kinase Inhibitor Therapy. *Research and Practice in Thrombosis and Haemostasis*.

[53] Maeda A., Tsuchida N., Uchiyama Y. (2024). Efficient Detection of Somatic UBA1 Variants and Clinical Scoring System Predicting Patients With Variants in VEXAS Syndrome. *Rheumatology*.

[54] Jachiet V., Kosmider O., Beydon M. (2025). Efficacy and Safety of Azacitidine for VEXAS Syndrome: A Large-Scale Retrospective Study From the FRENVEX Group. *Blood*.

[55] Aalbers A. M., van Daele P. L., Dalm V. A., Valk P. J., Raaijmakers M. H. (2024). Long‐Term Genetic and Clinical Remissions After Cessation of Azacitidine Treatment in Patients With VEXAS Syndrome. *HemaSphere*.

[56] Kataoka A., Mizumoto C., Kanda J. (2023). Successful Azacitidine Therapy for Myelodysplastic Syndrome Associated With VEXAS Syndrome. *International Journal of Hematology*.

