# Supplementary material for: JAK Inhibitors for Treatment of VEXAS Syndrome: A Systematic Review of 186 Cases
Source: Dermatol Res Pract. 2025 Sep 12;2025:9127126. doi: 10.1155/drp/9127126 (PMC12449113; doi:10.1155/drp/9127126)
Supplement: Supporting Information 1 — Supporting file 1 shows the keywords and query we used for this systematic search. [file 9127126.f1.docx]

**VEXAS & JAK. Inhibitors**

**July 6, 2024**

**PubMed: 173**

((“VEXAS”[Title/Abstract]) OR (“VEXAS”[MeSH Major Topic]) OR (“vacuoles, E1 enzyme, X-linked, autoinflammatory and somatic syndrome”[Title/Abstract]) OR (“VEXAS syndrome”[Title/Abstract]) OR (“vacuoles”[Title/Abstract]) OR (“vacuole”[Title/Abstract]) OR (“E1 enzyme”[Title/Abstract]) OR (“X-linked”[Title/Abstract]) OR (“UBA1 gene”[Title/Abstract]) OR (“Ubiquitin”[Title/Abstract]) OR (“somatic syndrome”[Title/Abstract]) OR (“somatic”[Title/Abstract]) OR (“somatic mutation”[Title/Abstract])) AND ((“Janus Kinase Inhibitors”[mh]) OR (Janus Kinase Inhibitors[tiab]) OR (Inhibitors, Janus Kinase[tiab]) OR (Kinase Inhibitors, Janus[tiab]) OR (JAK Inhibitors[tiab]) OR (Inhibitors, JAK[tiab]) OR (Janus Kinase Inhibitor[tiab]) OR (Inhibitor, Janus Kinase[tiab]) OR (Kinase Inhibitor, Janus[tiab]) OR (JAK Inhibitor[tiab]) OR (Inhibitor, JAK[tiab]) OR (JAK inhibitor[tiab]) OR (Janus kinase inhibitors[tiab]) OR (Janus tyrosine kinase inhibitor[tiab]) OR (Janus kinase inhibitor[tiab]) OR (cibinqo[tiab]) OR (pf 04965842[tiab]) OR (pf04965842[tiab]) OR (abrocitinib[tiab]) OR (incb 028050[tiab]) OR (incb 28050[tiab]) OR (incb028050[tiab]) OR (ly 3009104[tiab]) OR (ly3009104[tiab]) OR (olumiant[tiab]) OR (baricitinib[tiab]) OR (jte 052[tiab]) OR (jte 052a[tiab]) OR (jte052[tiab]) OR (leo 124249[tiab]) OR (leo124249[tiab]) OR (delgocitinib[tiab]) OR (fedratinib dihydrochloride[tiab]) OR (fedratinib dihydrochloride monohydrate[tiab]) OR (fedratinib hydrochloride[tiab]) OR (inrebic[tiab]) OR (sar302503[tiab]) OR (tg 101348[tiab]) OR (tg101348[tiab]) OR (fedratinib[tiab]) OR (filgotinib 2 butenedioate[tiab]) OR (filgotinib hydrochloride[tiab]) OR (filgotinib maleate[tiab]) OR (glpg 0634[tiab]) OR (glpg0634[tiab]) OR (gs 6034[tiab]) OR (gs6034[tiab]) OR (jyseleca[tiab]) OR (apoquel[tiab]) OR (oclacitinib maleate[tiab]) OR (oclacitinib[tiab]) OR (onx 0803[tiab]) OR (pacritinib citrate[tiab]) OR (pacritinib hydrochloride[tiab]) OR (sb 1518[tiab]) OR (sb1518[tiab]) OR (vonjo[tiab]) OR (pacritinib[tiab]) OR (asp015k[tiab]) OR (peficitinib hydrobromide[tiab]) OR (peficitinib[tiab]) OR (incb 18424[tiab]) OR (incb 424[tiab]) OR (incb018424[tiab]) OR (incb18424[tiab]) OR (jakafi[tiab]) OR (jakavi[tiab]) OR (kks 278[tiab]) OR (opzelura[tiab]) OR (ruxolitinib maleate[tiab]) OR (ruxolitinib phosphate[tiab]) OR (ruxolitinib[tiab]) OR (cgb 500[tiab]) OR (cgb500[tiab]) OR (cp 690 550[tiab]) OR (cp 690, 550[tiab]) OR (cp 690550[tiab]) OR (cp 690550 10[tiab]) OR (cp690 550[tiab]) OR (cp690, 550[tiab]) OR (cp690550[tiab]) OR (cp690550 10[tiab]) OR (pgn 600[tiab]) OR (tasocitinib[tiab]) OR (tasocitinib citrate[tiab]) OR (tofacitinib citrate[tiab]) OR (xeljanz[tiab]) OR (xeljanz xr[tiab]) OR (tofacitinib[tiab]) OR (abt 494[tiab]) OR (abt494[tiab]) OR (rinvoq[tiab]) OR (upadacitinib 2, 3 dihydroxybutanedioate[tiab]) OR (upadacitinib hemihydrate[tiab]) OR (upadacitinib hydrate[tiab]) OR (upadacitinib tartrate[tiab]) OR (upadacitinib[tiab]))

**WOS: 240**

((TS="VEXAS") OR (TS="vacuoles, E1 enzyme, X-linked, autoinflammatory and somatic syndrome") OR (TS="VEXAS syndrome") OR (TS="vacuoles") OR (TS="vacuole") OR (TS="E1 enzyme") OR (TS="X-linked") OR (TS="UBA1 gene") OR (TS="Ubiquitin") OR (TS="somatic syndrome") OR (TS="somatic") OR (TS="somatic mutation")) AND ((TS="Janus Kinase Inhibitors") OR (TS="Janus Kinase Inhibitors") OR (TS="Inhibitors, Janus Kinase") OR (TS="Kinase Inhibitors, Janus") OR (TS="JAK Inhibitors") OR (TS="Inhibitors, JAK") OR (TS="Janus Kinase Inhibitor") OR (TS="Inhibitor, Janus Kinase") OR (TS="Kinase Inhibitor, Janus") OR (TS="JAK Inhibitor") OR (TS="Inhibitor, JAK") OR (TS="JAK inhibitor") OR (TS="Janus kinase inhibitors") OR (TS="Janus tyrosine kinase inhibitor") OR (TS="Janus kinase inhibitor") OR (TS="cibinqo") OR (TS="pf 04965842") OR (TS="pf04965842") OR (TS="abrocitinib") OR (TS="incb 28050") OR (TS="incb028050") OR (TS="ly 3009104") OR (TS="ly3009104") OR (TS="olumiant") OR (TS="baricitinib") OR (TS="jte 052") OR (TS="jte 052a") OR (TS="jte052") OR (TS="leo 124249") OR (TS="leo124249") OR (TS="delgocitinib") OR (TS="fedratinib dihydrochloride") OR (TS="fedratinib dihydrochloride monohydrate") OR (TS="fedratinib hydrochloride") OR (TS="inrebic") OR (TS="sar302503") OR (TS="tg 101348") OR (TS="tg101348") OR (TS="fedratinib") OR (TS="filgotinib 2 butenedioate") OR (TS="filgotinib hydrochloride") OR (TS="filgotinib maleate") OR (TS="glpg 0634") OR (TS="glpg0634") OR (TS="gs 6034") OR (TS="gs6034") OR (TS="jyseleca") OR (TS="apoquel") OR (TS="oclacitinib maleate") OR (TS="oclacitinib") OR (TS="onx 0803") OR (TS="pacritinib citrate") OR (TS="pacritinib hydrochloride") OR (TS="sb 1518") OR (TS="sb1518") OR (TS="vonjo") OR (TS="pacritinib") OR (TS="asp015k") OR (TS="peficitinib hydrobromide") OR (TS="peficitinib") OR (TS="incb 18424") OR (TS="incb 424") OR (TS="incb018424") OR (TS="incb18424") OR (TS="jakafi") OR (TS="jakavi") OR (TS="kks 278") OR (TS="opzelura") OR (TS="ruxolitinib maleate") OR (TS="ruxolitinib phosphate") OR (TS="ruxolitinib") OR (TS="cgb 500") OR (TS="cgb500") OR (TS="cp 690 550") OR (TS="cp 690, 550") OR (TS="cp 690550") OR (TS="cp 690550 10") OR (TS="cp690 550") OR (TS="cp690, 550") OR (TS="cp690550") OR (TS="cp690550 10") OR (TS="pgn 600") OR (TS="tasocitinib") OR (TS="tasocitinib citrate") OR (TS="tofacitinib citrate") OR (TS="xeljanz") OR (TS="xeljanz xr") OR (TS="tofacitinib") OR (TS="abt 494") OR (TS="abt494") OR (TS="rinvoq") OR (TS="upadacitinib 2, 3 dihydroxybutanedioate") OR (TS="upadacitinib hemihydrate") OR (TS="upadacitinib hydrate") OR (TS="upadacitinib tartrate") OR (TS="upadacitinib"))

```

**Scopus: 763**

((TITLE-ABS-KEY("VEXAS")) OR (TITLE-ABS-KEY("vacuoles, E1 enzyme, X-linked, autoinflammatory and somatic syndrome")) OR (TITLE-ABS-KEY("VEXAS syndrome")) OR (TITLE-ABS-KEY("vacuoles")) OR

(TITLE-ABS-KEY("vacuole")) OR (TITLE-ABS-KEY("E1 enzyme")) OR

(TITLE-ABS-KEY("X-linked")) OR (TITLE-ABS-KEY("UBA1 gene")) OR

(TITLE-ABS-KEY("Ubiquitin")) OR (TITLE-ABS-KEY("somatic syndrome")) OR (TITLE-ABS-KEY("somatic")) OR (TITLE-ABS-KEY("somatic mutation"))) AND ((TITLE-ABS-KEY("Janus Kinase Inhibitors")) OR (TITLE-ABS-KEY("Janus Kinase Inhibitors")) OR (TITLE-ABS-KEY("Inhibitors, Janus Kinase")) OR (TITLE-ABS-KEY("Kinase Inhibitors, Janus")) OR (TITLE-ABS-KEY("JAK Inhibitors")) OR (TITLE-ABS-KEY("Inhibitors, JAK")) OR (TITLE-ABS-KEY("Janus Kinase Inhibitor")) OR (TITLE-ABS-KEY("Inhibitor, Janus Kinase")) OR (TITLE-ABS-KEY("Kinase Inhibitor, Janus")) OR (TITLE-ABS-KEY("JAK Inhibitor")) OR (TITLE-ABS-KEY("Inhibitor, JAK")) OR (TITLE-ABS-KEY("JAK inhibitor")) OR (TITLE-ABS-KEY("Janus kinase inhibitors")) OR (TITLE-ABS-KEY("Janus tyrosine kinase inhibitor")) OR (TITLE-ABS-KEY("Janus kinase inhibitor")) OR (TITLE-ABS-KEY("cibinqo")) OR (TITLE-ABS-KEY("pf 04965842")) OR (TITLE-ABS-KEY("pf04965842")) OR (TITLE-ABS-KEY("abrocitinib")) OR (TITLE-ABS-KEY("incb 028050")) OR (TITLE-ABS-KEY("incb 28050")) OR (TITLE-ABS-KEY("incb028050")) OR (TITLE-ABS-KEY("ly 3009104")) OR (TITLE-ABS-KEY("ly3009104")) OR (TITLE-ABS-KEY("olumiant")) OR (TITLE-ABS-KEY("baricitinib")) OR (TITLE-ABS-KEY("jte 052")) OR (TITLE-ABS-KEY("jte 052a")) OR (TITLE-ABS-KEY("jte052")) OR (TITLE-ABS-KEY("leo 124249")) OR (TITLE-ABS-KEY("leo124249")) OR (TITLE-ABS-KEY("delgocitinib")) OR (TITLE-ABS-KEY("fedratinib dihydrochloride")) OR (TITLE-ABS-KEY("fedratinib dihydrochloride monohydrate")) OR (TITLE-ABS-KEY("fedratinib hydrochloride")) OR (TITLE-ABS-KEY("inrebic")) OR (TITLE-ABS-KEY("sar302503")) OR (TITLE-ABS-KEY("tg 101348")) OR (TITLE-ABS-KEY("tg101348")) OR (TITLE-ABS-KEY("fedratinib")) OR (TITLE-ABS-KEY("filgotinib 2 butenedioate")) OR (TITLE-ABS-KEY("filgotinib hydrochloride")) OR (TITLE-ABS-KEY("filgotinib maleate")) OR (TITLE-ABS-KEY("glpg 0634")) OR (TITLE-ABS-KEY("glpg0634")) OR (TITLE-ABS-KEY("gs 6034")) OR (TITLE-ABS-KEY("gs6034")) OR (TITLE-ABS-KEY("jyseleca")) OR (TITLE-ABS-KEY("apoquel")) OR (TITLE-ABS-KEY("oclacitinib maleate")) OR (TITLE-ABS-KEY("oclacitinib")) OR (TITLE-ABS-KEY("onx 0803")) OR (TITLE-ABS-KEY("pacritinib citrate")) OR (TITLE-ABS-KEY("pacritinib hydrochloride")) OR (TITLE-ABS-KEY("sb 1518")) OR (TITLE-ABS-KEY("sb1518")) OR (TITLE-ABS-KEY("vonjo")) OR (TITLE-ABS-KEY("pacritinib")) OR (TITLE-ABS-KEY("asp015k")) OR (TITLE-ABS-KEY("peficitinib hydrobromide")) OR (TITLE-ABS-KEY("peficitinib")) OR (TITLE-ABS-KEY("incb 18424")) OR (TITLE-ABS-KEY("incb 424")) OR (TITLE-ABS-KEY("incb018424")) OR (TITLE-ABS-KEY("incb18424")) OR (TITLE-ABS-KEY("jakafi")) OR (TITLE-ABS-KEY("jakavi")) OR (TITLE-ABS-KEY("kks 278")) OR (TITLE-ABS-KEY("opzelura")) OR (TITLE-ABS-KEY("ruxolitinib maleate")) OR (TITLE-ABS-KEY("ruxolitinib phosphate")) OR (TITLE-ABS-KEY("ruxolitinib")) OR (TITLE-ABS-KEY("cgb 500")) OR (TITLE-ABS-KEY("cgb500")) OR (TITLE-ABS-KEY("cp 690 550")) OR (TITLE-ABS-KEY("cp 690, 550")) OR (TITLE-ABS-KEY("cp 690550")) OR (TITLE-ABS-KEY("cp 690550 10")) OR (TITLE-ABS-KEY("cp690 550")) OR (TITLE-ABS-KEY("cp690, 550")) OR (TITLE-ABS-KEY("cp690550")) OR (TITLE-ABS-KEY("cp690550 10")) OR (TITLE-ABS-KEY("pgn 600")) OR (TITLE-ABS-KEY("tasocitinib")) OR (TITLE-ABS-KEY("tasocitinib citrate")) OR (TITLE-ABS-KEY("tofacitinib citrate")) OR (TITLE-ABS-KEY("xeljanz")) OR (TITLE-ABS-KEY("xeljanz xr")) OR (TITLE-ABS-KEY("tofacitinib")) OR (TITLE-ABS-KEY("abt 494")) OR (TITLE-ABS-KEY("abt494")) OR (TITLE-ABS-KEY("rinvoq")) OR (TITLE-ABS-KEY("upadacitinib 2, 3 dihydroxybutanedioate")) OR (TITLE-ABS-KEY("upadacitinib hemihydrate")) OR (TITLE-ABS-KEY("upadacitinib hydrate")) OR (TITLE-ABS-KEY("upadacitinib tartrate")) OR (TITLE-ABS-KEY("upadacitinib")))

```

**Embase: 419**

(('VEXAS':ab,ti) OR ('vacuoles, E1 enzyme, X-linked, autoinflammatory and somatic syndrome':ab,ti) OR ('VEXAS syndrome':ab,ti) OR ('vacuoles':ab,ti) OR ('vacuole':ab,ti) OR ('E1 enzyme':ab,ti) OR ('X-linked':ab,ti) OR ('UBA1 gene':ab,ti) OR ('Ubiquitin':ab,ti) OR ('somatic syndrome':ab,ti) OR ('somatic':ab,ti) OR ('somatic mutation':ab,ti)) AND (('Janus Kinase Inhibitors':ab,ti) OR ('Janus Kinase Inhibitors':ab,ti) OR ('Inhibitors, Janus Kinase':ab,ti) OR ('Kinase Inhibitors, Janus':ab,ti) OR ('JAK Inhibitors':ab,ti) OR ('Inhibitors, JAK':ab,ti) OR ('Janus Kinase Inhibitor':ab,ti) OR ('Inhibitor, Janus Kinase':ab,ti) OR ('Kinase Inhibitor, Janus':ab,ti) OR ('JAK Inhibitor':ab,ti) OR ('Inhibitor, JAK':ab,ti) OR ('JAK inhibitor':ab,ti) OR ('Janus kinase inhibitors':ab,ti) OR ('Janus tyrosine kinase inhibitor':ab,ti) OR ('Janus kinase inhibitor':ab,ti) OR ('cibinqo':ab,ti) OR ('pf 04965842':ab,ti) OR ('pf04965842':ab,ti) OR ('abrocitinib':ab,ti) OR ('incb 028050':ab,ti) OR ('incb 28050':ab,ti) OR ('incb028050':ab,ti) OR ('ly 3009104':ab,ti) OR ('ly3009104':ab,ti) OR ('olumiant':ab,ti) OR ('baricitinib':ab,ti) OR ('jte 052':ab,ti) OR ('jte 052a':ab,ti) OR ('jte052':ab,ti) OR ('leo 124249':ab,ti) OR ('leo124249':ab,ti) OR ('delgocitinib':ab,ti) OR ('fedratinib dihydrochloride':ab,ti) OR ('fedratinib dihydrochloride monohydrate':ab,ti) OR ('fedratinib hydrochloride':ab,ti) OR ('inrebic':ab,ti) OR ('sar302503':ab,ti) OR ('tg 101348':ab,ti) OR ('tg101348':ab,ti) OR ('fedratinib':ab,ti) OR ('filgotinib 2 butenedioate':ab,ti) OR ('filgotinib hydrochloride':ab,ti) OR ('filgotinib maleate':ab,ti) OR ('glpg 0634':ab,ti) OR ('glpg0634':ab,ti) OR ('gs 6034':ab,ti) OR ('gs6034':ab,ti) OR ('jyseleca':ab,ti) OR ('apoquel':ab,ti) OR ('oclacitinib maleate':ab,ti) OR ('oclacitinib':ab,ti) OR ('onx 0803':ab,ti) OR ('pacritinib citrate':ab,ti) OR ('pacritinib hydrochloride':ab,ti) OR ('sb 1518':ab,ti) OR ('sb1518':ab,ti) OR ('vonjo':ab,ti) OR ('pacritinib':ab,ti) OR ('asp015k':ab,ti) OR ('peficitinib hydrobromide':ab,ti) OR ('peficitinib':ab,ti) OR ('incb 18424':ab,ti) OR ('incb 424':ab,ti) OR ('incb018424':ab,ti) OR ('incb18424':ab,ti) OR ('jakafi':ab,ti) OR ('jakavi':ab,ti) OR ('kks 278':ab,ti) OR ('opzelura':ab,ti) OR ('ruxolitinib maleate':ab,ti) OR ('ruxolitinib phosphate':ab,ti) OR ('ruxolitinib':ab,ti) OR ('cgb 500':ab,ti) OR ('cgb500':ab,ti) OR ('cp 690 550':ab,ti) OR ('cp 690, 550':ab,ti) OR ('cp 690550':ab,ti) OR ('cp 690550 10':ab,ti) OR ('cp690 550':ab,ti) OR ('cp690, 550':ab,ti) OR ('cp690550':ab,ti) OR ('cp690550 10':ab,ti) OR ('pgn 600':ab,ti) OR ('tasocitinib':ab,ti) OR ('tasocitinib citrate':ab,ti) OR ('tofacitinib citrate':ab,ti) OR ('xeljanz':ab,ti) OR ('xeljanz xr':ab,ti) OR ('tofacitinib':ab,ti) OR ('abt 494':ab,ti) OR ('abt494':ab,ti) OR ('rinvoq':ab,ti) OR ('upadacitinib 2, 3 dihydroxybutanedioate':ab,ti) OR ('upadacitinib hemihydrate':ab,ti) OR ('upadacitinib hydrate':ab,ti) OR ('upadacitinib tartrate':ab,ti) OR ('upadacitinib':ab,ti))

```

All: 1595

Duplicate: 396

Remained: 1199
