# Supplementary material for: JAK Inhibitors for Treatment of VEXAS Syndrome: A Systematic Review of 186 Cases
Source: Dermatol Res Pract. 2025 Sep 12;2025:9127126. doi: 10.1155/drp/9127126 (PMC12449113; doi:10.1155/drp/9127126)
Supplement: Supporting Information 2 — Supporting file 2 shows the quality assessment of included articles by the National Heart, Lung, and Blood Institute (NHLBI) quality assessment tools. [file 9127126.f2.docx]

Bourbon et al. 2021

| criteria | yes | no | Other (CD, NR, NA) |
| --- | --- | --- | --- |
| 1. Was the research question or objective in this paper clearly stated? | ++ |  |  |
| 2. Was the study population clearly specified and defined? | ++ |  |  |
| 3. Was the participation rate of eligible persons at least 50%? | ++ |  |  |
| 4. Were all the subjects selected or recruited from the same or similar populations (including the same time period)? Were inclusion and exclusion criteria for being in the study pre specified and applied uniformly to all participants? | ++ |  |  |
| 5. Was a sample size justification, power description, or variance and effect estimates provided? | ++ |  |  |
| 6. For the analyses in this paper, were the exposure(s) of interest measured prior to the outcome(s) being measured? |  | + | + |
| 7. Was the timeframe sufficient so that one could reasonably expect to see an association between exposure and outcome if it existed? | ++ |  |  |
| 8. For exposures that can vary in amount or level, did the study examine different levels of the exposure as related to the outcome (e.g., categories of exposure, or exposure measured as continuous variable)? |  | + | + |
| 9. Were the exposure measures (independent variables) clearly defined, valid, reliable, and implemented consistently across all study participants? | ++ |  |  |
| 10. Was the exposure(s) assessed more than once over time? |  | ++ |  |
| 11. Were the outcome measures (dependent variables) clearly defined, valid, reliable, and implemented consistently across all study participants? |  | + | + |
| 12. Were the outcome assessors blinded to the exposure status of participants? |  |  | ++ |
| 13. Was loss to follow-up after baseline 20% or less? | ++ |  |  |
| 14. Were key potential confounding variables measured and adjusted statistically for their impact on the relationship between exposure(s) and outcome(s)? |  | + | + |

| Quality rating (Good, Fair, or Poor) |
| --- |
| Rater 1: good |
| Rater 2: good |
| Additional comments (if poor please state why): |

Heiblig et al.(2)

| criteria | yes | no | Other (CD, NR, NA) |
| --- | --- | --- | --- |
| 1. Was the research question or objective in this paper clearly stated? | ++ |  |  |
| 2. Was the study population clearly specified and defined? | ++ |  |  |
| 3. Was the participation rate of eligible persons at least 50%? | ++ |  |  |
| 4. Were all the subjects selected or recruited from the same or similar populations (including the same time period)? Were inclusion and exclusion criteria for being in the study pre specified and applied uniformly to all participants? | ++ |  |  |
| 5. Was a sample size justification, power description, or variance and effect estimates provided? | ++ |  |  |
| 6. For the analyses in this paper, were the exposure(s) of interest measured prior to the outcome(s) being measured? | ++ |  |  |
| 7. Was the timeframe sufficient so that one could reasonably expect to see an association between exposure and outcome if it existed? | ++ |  |  |
| 8. For exposures that can vary in amount or level, did the study examine different levels of the exposure as related to the outcome (e.g., categories of exposure, or exposure measured as continuous variable)? |  | + | + |
| 9. Were the exposure measures (independent variables) clearly defined, valid, reliable, and implemented consistently across all study participants? | ++ |  |  |
| 10. Was the exposure(s) assessed more than once over time? |  | ++ |  |
| 11. Were the outcome measures (dependent variables) clearly defined, valid, reliable, and implemented consistently across all study participants? | + |  | + |
| 12. Were the outcome assessors blinded to the exposure status of participants? |  |  | ++ |
| 13. Was loss to follow-up after baseline 20% or less? | ++ |  |  |
| 14. Were key potential confounding variables measured and adjusted statistically for their impact on the relationship between exposure(s) and outcome(s)? | ++ |  |  |

2022

| Quality rating (Good, Fair, or Poor) |
| --- |
| Rater 1: good |
| Rater 2: good |
| Additional comments (if poor please state why): |

Casal Moura et al. (10)

2023

| criteria | yes | no | Other (CD, NR, NA) |
| --- | --- | --- | --- |
| 1. Was the research question or objective in this paper clearly stated? | ++ |  |  |
| 2. Was the study population clearly specified and defined? | ++ |  |  |
| 3. Was the participation rate of eligible persons at least 50%? | ++ |  |  |
| 4. Were all the subjects selected or recruited from the same or similar populations (including the same time period)? Were inclusion and exclusion criteria for being in the study pre specified and applied uniformly to all participants? | ++ |  |  |
| 5. Was a sample size justification, power description, or variance and effect estimates provided? |  | + | + |
| 6. For the analyses in this paper, were the exposure(s) of interest measured prior to the outcome(s) being measured? | ++ |  |  |
| 7. Was the timeframe sufficient so that one could reasonably expect to see an association between exposure and outcome if it existed? | ++ |  |  |
| 8. For exposures that can vary in amount or level, did the study examine different levels of the exposure as related to the outcome (e.g., categories of exposure, or exposure measured as continuous variable)? | ++ |  |  |
| 9. Were the exposure measures (independent variables) clearly defined, valid, reliable, and implemented consistently across all study participants? | ++ |  |  |
| 10. Was the exposure(s) assessed more than once over time? |  | ++ |  |
| 11. Were the outcome measures (dependent variables) clearly defined, valid, reliable, and implemented consistently across all study participants? | ++ |  |  |
| 12. Were the outcome assessors blinded to the exposure status of participants? |  |  | ++ |
| 13. Was loss to follow-up after baseline 20% or less? | ++ |  |  |
| 14. Were key potential confounding variables measured and adjusted statistically for their impact on the relationship between exposure(s) and outcome(s)? |  | + | + |

| Quality rating (Good, Fair, or Poor) |
| --- |
| Rater 1: good |
| Rater 2: good |
| Additional comments (if poor please state why): |

Gurnari et al. (12)

2023

| criteria | yes | no | Other (CD, NR, NA) |
| --- | --- | --- | --- |
| 1. Was the research question or objective in this paper clearly stated? |  | + | + |
| 2. Was the study population clearly specified and defined? | ++ |  |  |
| 3. Was the participation rate of eligible persons at least 50%? | ++ |  |  |
| 4. Were all the subjects selected or recruited from the same or similar populations (including the same time period)? Were inclusion and exclusion criteria for being in the study pre specified and applied uniformly to all participants? | ++ |  |  |
| 5. Was a sample size justification, power description, or variance and effect estimates provided? |  |  | ++ |
| 6. For the analyses in this paper, were the exposure(s) of interest measured prior to the outcome(s) being measured? | ++ |  |  |
| 7. Was the timeframe sufficient so that one could reasonably expect to see an association between exposure and outcome if it existed? | ++ |  |  |
| 8. For exposures that can vary in amount or level, did the study examine different levels of the exposure as related to the outcome (e.g., categories of exposure, or exposure measured as continuous variable)? |  | + | + |
| 9. Were the exposure measures (independent variables) clearly defined, valid, reliable, and implemented consistently across all study participants? |  | + | + |
| 10. Was the exposure(s) assessed more than once over time? |  |  | ++ |
| 11. Were the outcome measures (dependent variables) clearly defined, valid, reliable, and implemented consistently across all study participants? |  | + | + |
| 12. Were the outcome assessors blinded to the exposure status of participants? |  |  | ++ |
| 13. Was loss to follow-up after baseline 20% or less? |  |  | ++ |
| 14. Were key potential confounding variables measured and adjusted statistically for their impact on the relationship between exposure(s) and outcome(s)? |  |  | ++ |

| Quality rating (Good, Fair, or Poor) |
| --- |
| Rater 1: fair |
| Rater 2: fair |
| Additional comments (if poor please state why): |

Hadjadj et al. (18)

2024

| Quality rating (Good, Fair, or Poor) |
| --- |
| Rater 1: good |
| Rater 2: good |
| Additional comments (if poor please state why): |

| criteria | yes | no | Other (CD, NR, NA) |
| --- | --- | --- | --- |
| 1. Was the research question or objective in this paper clearly stated? | ++ |  |  |
| 2. Was the study population clearly specified and defined? | ++ |  |  |
| 3. Was the participation rate of eligible persons at least 50%? | ++ |  |  |
| 4. Were all the subjects selected or recruited from the same or similar populations (including the same time period)? Were inclusion and exclusion criteria for being in the study pre specified and applied uniformly to all participants? | ++ |  |  |
| 5. Was a sample size justification, power description, or variance and effect estimates provided? |  |  | ++ |
| 6. For the analyses in this paper, were the exposure(s) of interest measured prior to the outcome(s) being measured? |  | + | + |
| 7. Was the timeframe sufficient so that one could reasonably expect to see an association between exposure and outcome if it existed? | ++ |  |  |
| 8. For exposures that can vary in amount or level, did the study examine different levels of the exposure as related to the outcome (e.g., categories of exposure, or exposure measured as continuous variable)? |  | + | + |
| 9. Were the exposure measures (independent variables) clearly defined, valid, reliable, and implemented consistently across all study participants? | ++ |  |  |
| 10. Was the exposure(s) assessed more than once over time? |  | + | + |
| 11. Were the outcome measures (dependent variables) clearly defined, valid, reliable, and implemented consistently across all study participants? | + |  | + |
| 12. Were the outcome assessors blinded to the exposure status of participants? |  |  | ++ |
| 13. Was loss to follow-up after baseline 20% or less? | ++ |  |  |
| 14. Were key potential confounding variables measured and adjusted statistically for their impact on the relationship between exposure(s) and outcome(s)? | ++ |  |  |

Vitale et al. (25)

2025

| criteria | yes | no | Other (CD, NR, NA) |
| --- | --- | --- | --- |
| 1. Was the research question or objective in this paper clearly stated? | ++ |  |  |
| 2. Was the study population clearly specified and defined? |  |  | ++ |
| 3. Was the participation rate of eligible persons at least 50%? | ++ |  |  |
| 4. Were all the subjects selected or recruited from the same or similar populations (including the same time period)? Were inclusion and exclusion criteria for being in the study pre specified and applied uniformly to all participants? | ++ |  |  |
| 5. Was a sample size justification, power description, or variance and effect estimates provided? |  |  | ++ |
| 6. For the analyses in this paper, were the exposure(s) of interest measured prior to the outcome(s) being measured? | ++ |  |  |
| 7. Was the timeframe sufficient so that one could reasonably expect to see an association between exposure and outcome if it existed? | ++ |  |  |
| 8. For exposures that can vary in amount or level, did the study examine different levels of the exposure as related to the outcome (e.g., categories of exposure, or exposure measured as continuous variable)? |  |  | ++ |
| 9. Were the exposure measures (independent variables) clearly defined, valid, reliable, and implemented consistently across all study participants? | ++ |  |  |
| 10. Was the exposure(s) assessed more than once over time? |  | ++ |  |
| 11. Were the outcome measures (dependent variables) clearly defined, valid, reliable, and implemented consistently across all study participants? | + |  | + |
| 12. Were the outcome assessors blinded to the exposure status of participants? |  |  | ++ |
| 13. Was loss to follow-up after baseline 20% or less? | ++ |  |  |
| 14. Were key potential confounding variables measured and adjusted statistically for their impact on the relationship between exposure(s) and outcome(s)? |  |  | ++ |

| Quality rating (Good, Fair, or Poor) |
| --- |
| Rater 1: good |
| Rater 2: good |
| Additional comments (if poor please state why): |

Al-Hakim et al. (26)

2025

| criteria | yes | no | Other (CD, NR, NA) |
| --- | --- | --- | --- |
| 1. Was the research question or objective in this paper clearly stated? | ++ |  |  |
| 2. Was the study population clearly specified and defined? | ++ |  |  |
| 3. Was the participation rate of eligible persons at least 50%? | ++ |  |  |
| 4. Were all the subjects selected or recruited from the same or similar populations (including the same time period)? Were inclusion and exclusion criteria for being in the study pre specified and applied uniformly to all participants? | ++ |  |  |
| 5. Was a sample size justification, power description, or variance and effect estimates provided? | ++ |  |  |
| 6. For the analyses in this paper, were the exposure(s) of interest measured prior to the outcome(s) being measured? | ++ |  |  |
| 7. Was the timeframe sufficient so that one could reasonably expect to see an association between exposure and outcome if it existed? | ++ |  |  |
| 8. For exposures that can vary in amount or level, did the study examine different levels of the exposure as related to the outcome (e.g., categories of exposure, or exposure measured as continuous variable)? |  | + | + |
| 9. Were the exposure measures (independent variables) clearly defined, valid, reliable, and implemented consistently across all study participants? | ++ |  |  |
| 10. Was the exposure(s) assessed more than once over time? |  | ++ |  |
| 11. Were the outcome measures (dependent variables) clearly defined, valid, reliable, and implemented consistently across all study participants? | ++ |  |  |
| 12. Were the outcome assessors blinded to the exposure status of participants? |  |  | ++ |
| 13. Was loss to follow-up after baseline 20% or less? | ++ |  |  |
| 14. Were key potential confounding variables measured and adjusted statistically for their impact on the relationship between exposure(s) and outcome(s)? | ++ |  |  |

| Quality rating (Good, Fair, or Poor) |
| --- |
| Rater 1: good |
| Rater 2: good |
| Additional comments (if poor please state why): |

Wolff et al. (28)

2025

| criteria | yes | no | Other (CD, NR, NA) |
| --- | --- | --- | --- |
| 1. Was the research question or objective in this paper clearly stated? |  | + | + |
| 2. Was the study population clearly specified and defined? | ++ |  |  |
| 3. Was the participation rate of eligible persons at least 50%? | ++ |  |  |
| 4. Were all the subjects selected or recruited from the same or similar populations (including the same time period)? Were inclusion and exclusion criteria for being in the study pre specified and applied uniformly to all participants? | ++ |  |  |
| 5. Was a sample size justification, power description, or variance and effect estimates provided? | ++ |  |  |
| 6. For the analyses in this paper, were the exposure(s) of interest measured prior to the outcome(s) being measured? |  | + | + |
| 7. Was the timeframe sufficient so that one could reasonably expect to see an association between exposure and outcome if it existed? |  | + | + |
| 8. For exposures that can vary in amount or level, did the study examine different levels of the exposure as related to the outcome (e.g., categories of exposure, or exposure measured as continuous variable)? |  |  | ++ |
| 9. Were the exposure measures (independent variables) clearly defined, valid, reliable, and implemented consistently across all study participants? | ++ |  |  |
| 10. Was the exposure(s) assessed more than once over time? |  | ++ |  |
| 11. Were the outcome measures (dependent variables) clearly defined, valid, reliable, and implemented consistently across all study participants? | + |  | + |
| 12. Were the outcome assessors blinded to the exposure status of participants? |  |  | ++ |
| 13. Was loss to follow-up after baseline 20% or less? |  |  | ++ |
| 14. Were key potential confounding variables measured and adjusted statistically for their impact on the relationship between exposure(s) and outcome(s)? | ++ |  |  |

| Quality rating (Good, Fair, or Poor) |
| --- |
| Rater 1: good |
| Rater 2: fair |
| Additional comments (if poor please state why): |

Salehi et al. (15)

2023

| criteria | yes | no | Other (CD, NR, NA) |
| --- | --- | --- | --- |
| 1. Was the study question or objective clearly stated? |  |  | ++ |
| 2. Was the study population clearly and fully described, including a case definition? | ++ |  |  |
| 3. Were the cases consecutive? |  |  | ++ |
| 4. Were the subjects comparable? | ++ |  |  |
| 5. Was the intervention clearly described? | ++ |  |  |
| 6. Were the outcome measures clearly defined, valid, reliable and implemented consistently across all study participants? | ++ |  |  |
| 7. Was the length of follow up adequate? |  |  | ++ |
| 8. Were the statistical methods well described? | + |  | + |
| 9. Were the results well described? | ++ |  |  |

| Quality rating (Good, Fair, or Poor) |
| --- |
| Rater 1: fair |
| Rater 2: fair |
| Additional comments (if poor please state why): |

Diral et al. (16)

2023

| criteria | yes | no | Other (CD, NR, NA) |
| --- | --- | --- | --- |
| 1. Was the study question or objective clearly stated? |  | + | + |
| 2. Was the study population clearly and fully described, including a case definition? | ++ |  |  |
| 3. Were the cases consecutive? | ++ |  |  |
| 4. Were the subjects comparable? | ++ |  |  |
| 5. Was the intervention clearly described? | ++ |  |  |
| 6. Were the outcome measures clearly defined, valid, reliable and implemented consistently across all study participants? |  | + | + |
| 7. Was the length of follow up adequate? | ++ |  |  |
| 8. Were the statistical methods well described? |  |  | ++ |
| 9. Were the results well described? | ++ |  |  |

| Quality rating (Good, Fair, or Poor) |
| --- |
| Rater 1: fair |
| Rater 2: fair |
| Additional comments (if poor please state why): |

Kreutzinger et al. (19)

2024

| criteria | yes | no | Other (CD, NR, NA) |
| --- | --- | --- | --- |
| 1. Was the study question or objective clearly stated? | ++ |  |  |
| 2. Was the study population clearly and fully described, including a case definition? | ++ |  |  |
| 3. Were the cases consecutive? |  |  | ++ |
| 4. Were the subjects comparable? |  |  |  |
| 5. Was the intervention clearly described? | ++ |  |  |
| 6. Were the outcome measures clearly defined, valid, reliable and implemented consistently across all study participants? |  | + | + |
| 7. Was the length of follow up adequate? | ++ |  |  |
| 8. Were the statistical methods well described? | ++ |  |  |
| 9. Were the results well described? | ++ |  |  |

| Quality rating (Good, Fair, or Poor) |
| --- |
| Rater 1: fair |
| Rater 2: fair |
| Additional comments (if poor please state why): |

Alamo et al. (24)

2024

| criteria | yes | no | Other (CD, NR, NA) |
| --- | --- | --- | --- |
| 1. Was the study question or objective clearly stated? | ++ |  |  |
| 2. Was the study population clearly and fully described, including a case definition? | ++ |  |  |
| 3. Were the cases consecutive? |  |  | ++ |
| 4. Were the subjects comparable? | ++ |  |  |
| 5. Was the intervention clearly described? | ++ |  |  |
| 6. Were the outcome measures clearly defined, valid, reliable and implemented consistently across all study participants? |  | + | + |
| 7. Was the length of follow up adequate? | ++ |  |  |
| 8. Were the statistical methods well described? | ++ |  |  |
| 9. Were the results well described? | ++ |  |  |

| Quality rating (Good, Fair, or Poor) |
| --- |
| Rater 1: good |
| Rater 2: good |
| Additional comments (if poor please state why): |
